# Uncovering the Intricate and Heterogeneous Cellular Microenvironment of Cutaneous Melanoma

**DOI:** 10.3390/medicina62040739

**Published:** 2026-04-13

**Authors:** Dana Antonia Țăpoi, Ioana Maria Lambrescu, Catalin Gabriel Manole, Gisela Gaina, Laura Cristina Ceafalan

**Affiliations:** 1 Department of Cellular and Molecular Biology and Histology, “Carol Davila” University of Medicine and Pharmacy, 020021 Bucharest, Romania; 2 “Victor Babeș” National Institute of Pathology, 050096 Bucharest, Romania

**Keywords:** cutaneous melanoma, microenvironment, therapy, extracellular matrix

## Abstract

*Background and Objectives*: Cutaneous melanoma (CM) is one of the most aggressive skin malignancies due to its rapid progression and high therapeutic resistance. Growing evidence demonstrates that the tumor microenvironment (TME)—comprising diverse immune, stromal, vascular, and epidermal cell populations alongside various cytokines and growth factors, as well as extracellular matrix (ECM) components—plays a crucial role in tumor heterogeneity, metastatic potential, and response to therapy. This review aims to synthesise current knowledge on the cellular and non-cellular constituents of the CM microenvironment and clarify their contributions to tumor progression, immune evasion, and treatment resistance. *Materials and Methods*: We conducted a narrative review of recent experimental, clinical, and translational studies investigating melanoma–microenvironment interactions, integrating evidence from in vitro, in vivo, and human tissue analyses. *Results*: Melanoma exhibits marked intra-tumoral heterogeneity driven by genetic, epigenetic, and microenvironmental influences. Cancer-associated fibroblasts, adipocytes, endothelial cells, and keratinocytes are reprogrammed by melanoma cells to promote invasion, angiogenesis, and metastasis. Immune subsets play divergent roles: neutrophils, M2 macrophages, myeloid-derived suppressor cells, and tolerogenic dendritic cells foster immune suppression, while lymphocytes—particularly CD8^+^ T cells, TFH cells, and B cells —are associated with improved outcomes but often become dysfunctional. ECM remodeling, including collagen deposition, integrin signaling, and increased matrix stiffness, actively remodels the tissue to support tumor growth and immune evasion. Hypoxia-inducible factor (HIF)-mediated signaling drives cell dedifferentiation, angiogenesis, and metabolic changes that contribute to treatment resistance. Consequently, emerging therapeutic strategies are moving beyond targeting tumor cells alone to focus on modulating TME components, counteracting immunosuppression, hypoxia, metabolic reprogramming, and extracellular vesicle signaling. *Conclusions*: The TME profoundly modulates tumor behavior and therapeutic response. A deeper understanding of the reciprocal interactions between melanoma cells and their microenvironmental components may enable the development of more effective strategies for early detection, prognosis, and personalized therapies.

## 1. Introduction

Cutaneous melanoma (CM) is a highly aggressive malignancy, responsible for the vast number of skin-cancer-related deaths [1,2]. Although it represents about 4% of all skin cancers, CM remains a significant public health concern due to its disproportionate lethality relative to its incidence, being responsible for more than 75% of skin cancer-related deaths [3,4].

To date, Breslow depth of invasion remains the most important prognostic factor, as thick CM portends the worst outcomes [5]. Early detection of melanoma remains paramount, and prognosis is closely linked to stage at diagnosis [6,7]. While the advent of immune checkpoint inhibitors (ICIs) and BRAF/MEK targeted therapies has transformed the landscape of advanced disease, patients often develop primary or acquired resistance to these treatments. Current prognostic tools, such as the Breslow depth and AJCC staging, are essential but often insufficient to predict which patients will experience early metastasis or fail frontline immunotherapy. Emerging evidence suggests that these clinical gaps are driven not only by the tumor’s genetic profile but by the intricate and heterogeneous tumor microenvironment (TME), which orchestrates immune escape and metabolic reprogramming.

The melanoma microenvironment represents a heterogeneous entity composed of both cellular components, including tumor and immune cells, and non-cellular components such as the extracellular matrix (ECM) and various cytokines and growth factors, all of which influence tumor behavior [8]. Consequently, this review provides a comprehensive map of the melanoma TME to assist clinicians and researchers in identifying actionable targets and prognostic biomarkers. We first explore how intra-tumoral heterogeneity and cellular communication drive drug resistance. We then categorize the cellular components of the TME—including cancer-associated fibroblasts (CAFs), endothelial cells, and diverse immune subsets—detailing their specific roles in promoting tumor progression versus anti-tumor immunity. Finally, we synthesize these mechanistic insights into a discussion of emerging clinical strategies, focusing on how modulating the TME may overcome current barriers in immunotherapy and targeted treatments.

## 2. Materials and Methods

We conducted a narrative review of the literature to synthesize current evidence on the CM TME. A comprehensive search was performed using the PubMed, Scopus, and Google Scholar databases. While we prioritized high-impact research published between 2010 and 2025, including original experimental studies (in vitro and in vivo), translational analyses of human tissue, and clinical trials, to ensure clinical relevance, some older foundational studies and seminal works were also included to provide essential historical context on melanoma pathogenesis.

The search strategy utilized combinations of the following keywords: “cutaneous melanoma,” “tumor microenvironment,” “cancer-associated fibroblasts (CAFs),” “stromal cells”, “immune cells”, “growth factors”, “cytokines”, “extracellular matrix remodeling”, “tumor heterogeneity”, “metabolic reprogramming”, “hypoxia”, “immune checkpoint inhibitors” and “therapeutic resistance”. Articles were selected based on their relevance to the cellular and non-cellular components of the melanoma niche and their contribution to understanding patient outcomes and drug resistance.

## 3. Intra-Tumoral Heterogeneity

Tumor heterogeneity is defined as the phenotypic and biological variation among cell subpopulations within a single tumor (intra-tumoral), between different tumors of the same histological subtype in one patient (inter-tumoral), or among different patients (inter-patient) [9]. As a result, differences in sensitivity to anticancer drugs arise from genetic, transcriptomic, epigenetic, and/or phenotypic alterations [10]. In addition to these alterations, pressure from the host immune system is undoubtedly one of the factors influencing tumor heterogeneity [11,12]. Furthermore, the TME influences cellular communication by altering signaling between different cell compartments, thereby contributing to drug resistance alongside mutational and non-mutational mechanisms within tumor cells [13].

Melanoma is a particularly heterogeneous cancer, exhibiting a wide range of genetic changes and expressing a variety of molecular markers [13]. According to a new study investigating melanoma patients with primary CM and concomitant metastases, the TME changes as the tumor progresses. Therefore, the immune cell profile alone may be insufficient to diagnose individuals with primary melanoma and predict their prognosis [14]. Thus, in the following sections, we examine the principal components of the TME in melanoma to elucidate the role each element plays in disease progression and in the emergence of treatment resistance.

## 4. Components of the TME

As stated above, the melanoma microenvironment comprises both cellular and non-cellular components, whose complex interactions are crucial for tumor progression.

### 4.1. Cellular Components

The microenvironment’s cellular components include immune cells, cancer-associated fibroblasts (CAFs), cancer-associated adipocytes, endothelial cells and keratinocytes that interact with melanoma cells to promote tumour progression [8,15].

Melanoma cells alter the TME in various ways. For example, they may stimulate CAFs, especially in metastatic conditions, increasing invasiveness and angiogenesis [16]. Similarly, melanoma cells can induce tumor-promoting macrophage M2 polarization [17]. Moreover, melanoma cells have been shown to elicit a fibroblast-like phenotype in adipocytes, therefore promoting tumor development [18]. Keratinocytes may be activated by melanoma cells to promote invasiveness [16]. In addition, melanoma cells release extracellular vesicles containing various microRNAs and proteins that can promote cell proliferation and invasiveness, tumour angiogenesis and resistance to therapy, as well as alter immune cells’ function [4,19,20,21,22,23]. Specifically, melanoma cells can hinder the maturation and function of dendritic cells, which in turn reduces the capability of these cells to present antigens [24].

In acidic environments, melanoma cells can adopt a stem cell phenotype and express characteristic markers [25]. Melanoma stem cells can self-renew indefinitely and differentiate into various cell types, thus contributing to the highly heterogeneous cellular microenvironment and promoting invasiveness, metastases and resistance to therapy [26,27].

#### 4.1.1. Stromal Cells

##### Cancer-Associated Fibroblasts (CAFs) Are Key Regulators in Melanoma Progression

Fibroblasts are mostly activated by epigenetic changes driven by cytokines and growth factors produced by melanoma cells. CAFs may originate from multiple cell types, including resident fibroblasts, bone marrow-derived mesenchymal stem cells, macrophages, adipocytes, epithelial and endothelial cells, or pericytes [15]. These activated fibroblasts display unique immunohistochemical, molecular, and genetic profiles. One hallmark of CAFs is the overexpression of α-SMA, similar to myofibroblasts involved in wound healing [28,29,30], alongside FAP (fibroblast activation protein), vimentin, FSP1 (fibroblast-specific protein 1), and PDGFR-α and β (platelet-derived growth factor receptor) [31]. Normal fibroblasts secrete cytokines and inhibitory factors (e.g., interleukin 6—IL-6, interleukin 15—IL-15, transforming growth factor-beta—TGF-β, pigment epithelium-derived factor—PEDF) that can suppress melanoma growth, but factors like IL-6 and TGF-β can act as tumor suppressors early but shift to pro-tumorigenic roles in advanced disease [32].

CAFs actively remodel the ECM, enhancing melanoma cell migration, survival, and resistance to therapy, and modulating immune responses via factors such as matrix metalloproteinases (MMPs), interleukin-1α (IL-1α), interleukin-1β (IL-1β), interleukin-6, and interleukin-8 (IL-8). Additional growth factors include hepatocyte growth factor (HGF), stem cell factor (SCF), basic fibroblast growth factor (b-FGF), vascular endothelial growth factor (VEGF), and insulin growth factor-1 (IGF-1) [15,16,29]. Melanoma cells co-cultured with CAFs or conditioned media from CAFs exhibit enhanced invasion and migration. In mouse models, β-catenin suppression in CAFs reduced tumor-driven vascularization, underscoring their importance in melanoma progression [33,34]. CAFs, as well as melanoma cells, express TRAF6 (TNF Receptor Associated Factor 6), which mediates reciprocal signaling that promotes tumor progression by driving CAF activation, which in turn enhances melanoma invasiveness and drug resistance [31]. Melanoma–CAF interactions also contribute to therapy resistance, notably through fibroblast-derived HGF, which activates MET signaling in melanoma cells and reactivates MAPK and PI3K pathways, driving resistance to BRAF inhibitors. Elevated stromal HGF levels in BRAF-mutant patients correlated with poor prognosis and reduced treatment response [35]. Furthermore, CAFs contribute to immune evasion by expressing PD-L1/PD-L2 and secreting C-X-C Motif Chemokine Ligand 5 (CXCL5) [29,36,37]. Direct fibroblast–melanoma cell contact is crucial for tumor cell survival, as fibroblast elimination or conditioned media alone fail to support long-term growth [38].

Fibroblasts also shape tumor architecture—co-cultures with fibroblasts form compact 3D spheroids resembling in vivo tumors and remodel collagen fibers to generate aligned, thickened matrices [39]. Matrix CAFs (mCAFs), immunomodulatory CAFs (iCAFs), myofibroblast-like CAFs (myoCAFs), and unclassifiable CAFs (ucCAFs) are the four subtypes of CAFs that have been found in skin tumors due to the research conducted by Forsthuber and colleagues. Therefore, mCAFs build thick collagen networks that serve as physical barriers to immune infiltration. iCAFs drive immune evasion via immunosuppressive regulators. MyoCAFs are present in all skin malignancies and are thought to be involved in fibrosis and perivascular remodeling. On the other hand, ucCAFs are not well understood [40].

Directly targeting CAF is currently in the early stages of clinical translation for therapeutic purposes. A possible therapeutic target is fibroblast activation protein (FAP), a membrane-bound serine protease that is specifically produced by activated stromal fibroblasts in the tumor microenvironment [41]. A phase Ib study evaluated the FAP-targeted immunocytokine simlukafusp alfa (FAP-IL2v) in combination with pembrolizumab in patients with advanced or metastatic melanoma. The therapy demonstrated satisfactory tolerability and was associated with preferential expansion of NK and CD8^+^ T cells. Nevertheless, antitumor efficacy was limited in individuals who had been pretreated with checkpoint inhibitors [42].

All these findings point to the potential clinical significance of CAF-rich melanomas for both biomarker-guided stratification and future combination treatments intended to overcome immune exclusion and treatment resistance.

*CAFs are primary drivers of the physical and biochemical barriers to treatment. They actively drive melanoma progression by remodeling the ECM and secreting factors like chemokines and HGF, which physically exclude T cells and induce resistance to targeted therapies. High stromal FAP or HGF expression serves as a negative prognostic indicator and a biomarker for potential resistance to BRAF inhibitors. Therapeutic strategies targeting the CAF-mediated chemokine release or HGF/MET signaling are currently high-priority areas for overcoming resistance in clinical trials.*

##### The Role of Adipocytes in Melanoma Progression

Although the role of adipocytes in melanoma formation is still unclear, these cells appear to change their phenotype to resemble fibroblasts and drive tumor progression by remodeling the ECM [43]. Regarding cutaneous melanomas, adipocytes shift towards a fibroblast-like phenotype and increase melanoma cell migration via the β-catenin and LEF-1 pathways [44]. Interestingly, the aggressiveness of melanomas increases as the quantity of adipose tissue surrounding the tumour increases [45], and multiple murine models demonstrated that obesity is positively correlated with melanoma progression [45,46,47]. Furthermore, adipocytes have been shown to enhance tumor progression by transferring lipids to melanoma cells through FATP lipid transporters [48]. Additionally, adipocytes produce various growth factors and cytokines such as fibroblast growth factor-21 (FGF-21), hepatocyte growth factor (HGF), IGF-1, VEGF, insulin-like growth factor-binding protein (IGFBP), IL-6, interleukin 11 (IL-11), and tumor necrosis factor-alpha (TNF-α), that stimulate melanoma cell proliferation and resistance to therapy [49]. Adipocytes also promote immune escape in melanoma by expressing high levels of PD-L1 [50]. A recent in vitro study demonstrated that preadipocytes promote M2 polarization in macrophages, melanoma progression, and metastases [51].

Adipocyte-rich melanoma microenvironment is increasingly recognized for its therapeutic significance, both as possible treatment response modifiers and as metabolic promoters of tumor development. For instance, fatty acid uptake is a potential therapeutic target as pharmacologic suppression of these mechanisms could decrease melanoma growth and invasion [48,52]. Furthermore, researchers are now exploring whether traits related to obesity can better stratify patients for immunotherapy [53]. In a recent multicenter cohort, melanoma patients on first-line anti-PD-1 ± anti-CTLA-4 treatment had their BMI and computed tomography (CT)-derived body composition characteristics specifically assessed. The study concluded that a lower visceral adipose tissue index and greater skeletal muscle density were associated with better overall survival, whereas participants with an underweight BMI had worse progression-free survival [53].

*Adipocytes actively fuel melanoma progression by acting as a local energy source through lipid transfer and by secreting a “secretome” of inflammatory cytokines (IL-6, TNF-α) and growth factors (HGF, IGF-1). Their ability to adopt a fibroblast-like phenotype and express high levels of PD-L1 makes them significant contributors to both metabolic reprogramming and immune escape in the cutaneous niche.*

#### 4.1.2. Endothelial Cells

Novel blood vessel formation is a key process in tumour spread [54]. Several different processes, including sprouting angiogenesis, intussusceptive angiogenesis, vascular co-option, vasculogenic mimicry, and vasculogenesis, might be responsible for this phenomenon [55]. Among the multiple factors involved in vasculogenesis in CM, endovascular progenitor cells play a crucial role as they infiltrate the tumour bed early on and subsequently form new blood vessels in areas previously devoid of vascular structures. Importantly, this phenomenon develops independently of VEGF signaling, which may explain the reduced efficacy of targeting VEGF in treating cutaneous melanomas [56]. Nevertheless, in vitro studies also demonstrated that endothelial cells interact with the ECM, activating integrin signaling and subsequently VEGFR2, thus promoting angiogenesis [57]. In addition, endothelial cells interact with the ECM by expressing collagens that bind to CD93 or integrin receptors on tumor cells [58]. Consequently, murine models showed that blocking CD93 promoted normal vascular formation, reduced tumor growth, and increased response to chemotherapy [59]. Additionally, endothelial cells influence immune cells in the melanoma microenvironment [55]. These cells are the primary source of type 1 IFN, which attracts and activates CD8^+^ antitumor cells. This route may be targeted to boost immune cells’ antitumor activity. [60].

Finally, endothelial cells can also shift their phenotype towards a fibroblast-like state. Melanoma cells derived TGF-β1 induces endothelial-to-mesenchymal transition, which in turn promotes metastases [8,61]. While displaying mesenchymal markers, including fibroblast-specific protein 1 (FSP1) and alpha-smooth muscle actin (α-SMA), these altered endothelial cells exhibit reduced expression of conventional vascular markers, such as CD31 [62]. Importantly, decreased expression of ERG and FLI1 transcription factors in endothelial cells is associated with decreased survival in melanoma patients [63].

#### 4.1.3. Immune Cells

##### Neutrophils Also Influence the Behavior of CM

Elevated neutrophil counts in peripheral blood are associated with decreased survival in melanoma patients [64]. In this context, the presence of neutrophils, as indicated by CD66b immunostaining in melanoma tissue samples, is an independent negative prognostic factor [65]. The pro-tumoral action of neutrophils is mostly mediated by N2 neutrophils, which are polarized by cancer stem cells [66]. Neutrophils are recruited to the TME through various signals, including ultraviolet radiation-induced high-mobility group box 1 HMGB1 release from epidermal keratinocytes and recruitment via melanoma-derived CXCL5 [66,67,68,69]. In this setting, an abundant neutrophilic infiltrate is associated with a high risk for metastases in murine models [67]. Furthermore, neutrophil extracellular traps (NETs) affect endothelial barrier stability, thereby driving angiogenic responses and enabling metastatic spread [17,70]. In metastatic melanoma, neutrophils also show heightened annexin A1 expression, which facilitates tumour development via pathways regulated by formyl peptide receptors (FPRs) [71].

*Elevated peripheral and intratumoral neutrophils are independent negative prognostic factors in melanoma. Their pro-tumoral role is mediated by N2-polarization and the formation of neutrophil Extracellular Traps (NETs), which disrupt the endothelial barrier to promote metastatic spread. In this context, the neutrophil-to-lymphocyte ratio (NLR) serves as a cost-effective, easily accessible blood-based biomarker for predicting decreased survival.*

##### Tumor-Associated Macrophages (TAMs)

TAMs are the most abundant inflammatory cell population in the TME and exert important pro-tumoral roles in CM [8,72]. Several immunohistochemical studies demonstrated that extensive TAM infiltration in the primary tumor is associated with locally advanced tumors, lympho-vascular invasion, recurrence, metastasis and decreased survival [73,74]. On the contrary, other studies found no association between macrophage density and melanoma prognosis [8]. This ambivalent behavior may be explained by the polarization phenomenon, which transforms TAMs into either M1 anti-tumoral or M2 pro-tumoral macrophages, each subtype producing various cytokines that influence neoplastic growth [75]. In this context, M1 macrophages release pro-inflammatory cytokines such as interleukin 12 (IL-12), interleukin 23 (IL-23), and TNF-α, while M2 macrophages produce mostly IL-6, IL-8, VEGFA, and VEGFC. For instance, murine models showed that macrophage-derived TNF-α contributes to resistance to MAPK inhibitors in *BRAF*-mutated melanomas [76]. Additionally, TAMs from patients with metastatic disease exhibited significantly higher levels of CCL20, TNF-α, and VEGFA. [77]. The polarization and survival of M2 macrophages is driven by miR-125b-5p secreted by melanoma cells into the microenvironment [23]. Lastly, the pro-tumoral effects of TAMs are enhanced by direct contact between melanoma cells and TAMs. According to Pizzuro et al., TAMs exhibit different morphologies within the TME: those located closer to tumor cells typically take on an elongated shape with extended protrusions that improve cell-to-cell interactions, while macrophages located farther away remain rounder and smaller [39]. In this context, melanoma cells stimulate PD-L1 and V-domain Ig suppressor of T cell activation (VISTA) expression on M2-TAMs, which directly inhibits T-cell cytotoxicity [78,79]. Elevated VISTA levels correlate with poorer survival in primary melanoma and foster a protumor microenvironment by increasing Treg infiltration and enhancing PD-L1 expression on macrophages [79,80].

TAMs are increasingly recognized for their therapeutic significance, serving not only as mediators of immune suppression but also as potential indicators for immunotherapy response. According to recent studies, metastatic melanoma patients who did not respond to anti-PD-1 treatment tended to have a higher concentration of CD16^−^ M2-like macrophages [81]. In contrast, myeloid cell signatures indicative of inflammatory monocyte/macrophage influx were associated with better overall and progression-free survival, as well as a treatment response [81]. Most importantly, not every macrophage subgroup is detrimental to clinical outcomes. Macrophage phenotyping could improve patient classification beyond total TAM density alone, since intratumoral CD16^+^ macrophages have been linked to better outcomes in patients receiving combination anti-PD-1 and anti-CTLA-4 treatment [82]. Recent findings suggest that CCR1^+^ macrophages are linked to resistance to immune checkpoint inhibition in melanoma. These data reinforce the idea that certain TAM niches, rather than the sheer number of macrophages, may predict treatment failure [83]. Collectively, this evidence has direct translational significance, as macrophage-targeted therapies are now undergoing clinical trials, mostly in combination with immune checkpoint inhibitors rather than as standalone treatments [84]. CSF1R (colony-stimulating factor 1 receptor), a crucial regulator of macrophage recruitment, survival, and polarisation within the tumor microenvironment, is being targeted in early-phase clinical studies for melanoma [85].

*TAMs are the most abundant inflammatory population in the melanoma TME. Their pro-tumoral role is largely driven by M2-polarization, which facilitates immune evasion via PD-L1 expression and promotes therapeutic resistance, making the M2/M1 ratio a vital prognostic indicator. Targeting the recruitment and polarization of TAMs represents a major clinical strategy for re-sensitizing tumors to immunotherapy.*

##### Tumor Infiltrating Lymphocytes (TILs)

First described by Clark, melanoma TILs can be divided into intratumoral and peritumoral lymphocytes [86]. The initial system for reporting TILs in melanoma, proposed by Clark, included the following categories: absent, brisk, and non-brisk [87]. Subsequently, the Melanoma Institute Australia developed a scoring system for grading TILs ranging from 0 to 3 as follows: absent (0), mild multifocal or a mild/moderate focal infiltrate (1), moderate or marked multifocal, a marked focal or a mild diffuse TIL pattern (2) and a moderate or marked diffuse infiltrate (3) [88]. Increased TILs, as evaluated by either score, have been associated with a better prognosis [86,88,89,90]. Nevertheless, some studies found no association between TILs density and melanoma prognosis [4,91,92]. In this context, it appears that in addition to the quantity of TILs, their quality is also crucial for predicting prognosis in CM. Phenotypically, lymphocytes can be divided based on immunohistochemical expression into CD20^+^ B cells, CD4^+^ helper T cells, CD8^+^ cytotoxic T cells, NK cells, and FOXP3^+^ Regulatory T cells (Treg) [86,90].

To begin with, CD4^+^ helper T cells can be further divided into Th1, Th2, and Th17 cells and follicular helper T cells (Tfh). In this respect, Th1 generally play an anti-tumor role by producing IFNγ, enhancing CD8^+^ cytotoxic lymphocyte activity, and recruiting NK cells and M1-polarised macrophages [93]. However, Th2 cells have more complex roles in cancer development. By generating interleukin 4 (IL-4) and attracting eosinophils, which have been shown to eradicate metastatic melanoma in lung samples, these cells may have anti-tumor action [94]. Th2 cells also produce interleukin 5 (IL-5), which may accelerate melanoma development [95].

Th17 are mostly involved in anti-microbial responses, but their signature cytokine, interleukin 17 (IL-17), may also influence tumor behavior [93]. In vitro studies showed that Th17 are superior to Th1 in eradicating melanoma cells by producing IFNγ and IL-17, recruiting dendritic cells, and activating tumor-specific CD8^+^ T cells [96,97]. Nevertheless, Th17 have also been implicated in melanoma progression by promoting angiogenesis [15]. Finally, Tfh cells also influence melanoma behavior in various ways. Tfh are characterized by chemokine receptor 5 (CXCR5) and PD-1 expression and are vital for the formation of tertiary lymphoid structures (TLS), which correlate with enhanced patient survival [93,98].

Finally, Tregs represent CD4^+^ lymphocytes that express the FoxP3 transcription factor and are involved in both maintaining physiological immune tolerance and creating an immunosuppressive microenvironment in tumour tissue [93,99]. Melanoma cells promote the recruitment and activation of Tregs [100,101], and in turn, Tregs promote melanoma progression by enhancing immune evasion [102]. Tregs also produce adenosine, which suppresses the activity of dendritic cells and induces a state of immunosuppression in effector T lymphocytes [103]. Therapeutic depletion of Tregs via anti-CTLA-4 [104] or targeting the adenosine pathway represents a key clinical strategy to restore the anti-tumor immune response.

CD8^+^ T lymphocytes are the principal effectors of antitumor immunity, eliminating cancer cells via cytokine release (IFN-γ, TNF-α) and direct cytotoxicity through granzyme- and perforin-containing exosomes [105]. Multipronged T-cell responses, recognizing multiple tumor-associated epitopes, can enhance cytotoxicity, potentially via molecular mimicry [106]. Nevertheless, CD8^+^ cells frequently become exhausted due to ongoing exposure to melanoma antigens, resulting in upregulation of inhibitory checkpoint molecules and a decrease in cytotoxic activity. Following the uncovering of this mechanism, immune checkpoint inhibitors were developed. Anti-PD1 and anti-CTLA-4 therapy is used to restore exhausted CD8^+^ lymphocytes, and its administration has significantly increased melanoma survival [107,108].

Similarly, in advanced melanoma, a subset of T cells coexpressing PD-1 and Tim-3 (mucin domain-containing molecule 3) has been identified, with Tim-3 blockade partially restoring T-cell function and enhancing antitumor activity [109].

The same group also reported TIGIT as another inhibitory receptor, whose inhibition, when combined with PD-1, may similarly reverse T-cell dysfunction [110]. Furthermore, loss of PTEN function, a tumour suppressor gene frequently mutated in melanoma, reduces the recruitment of cytotoxic cells and their anti-tumour activity, promoting resistance to immunotherapy [111].

*The balance between “brisk” CD8^+^ T-cell infiltration and immunosuppressive FoxP3^+^ Tregs is the primary determinant of immunotherapy success. The presence of specialized CD4^+^ (Tfh) cells is further essential for the formation of protective tertiary lymphoid structures (TLS).*

B lymphocytes are generally regarded as anti-tumoral due to their ability to promote cytotoxicity, produce antibodies, and act as antigen-presenting cells [112]. These cells can form TLS, which has been associated with improved outcomes in various cancers [15,113]. In melanoma, B cells enhance responses to ICIs by facilitating CD8^+^ T-cell recruitment and the formation of TLS. Notably, the co-occurrence of CD20^+^ B cells and CD8^+^ T cells is associated with improved survival, and B-cell gene signatures are enriched in tumors of ICI responders [113,114,115,116]. This theory has been validated by several immunohistochemistry studies of CM, which show that higher B cell counts in tumor tissue are associated with better survival [117,118,119]. Similarly, mRNA sequencing data analysis showed that high expression of the B cell signature is associated with improved survival [120]. Furthermore, iatrogenic depletion of B cells increases melanoma growth in murine models [121]. Nevertheless, B cells can also promote angiogenesis, chronic inflammation, and resistance to MAPK-targeted therapy [122]. Similarly, increased numbers of CD138^+^ plasma cells in melanoma tissue samples have been associated with decreased survival, but due to the limited number of studies, the exact mechanism remains unknown; however, it may involve oligoclonal IgA production [123,124].

*Beyond antibody production, B cells are vital for organizing the immune response within TLS. A strong B-cell/TLS signature is currently one of the most reliable predictors of long-term survival and response to immune checkpoint blockade.*

NK lymphocytes are innate cytotoxic cells that can identify and eradicate melanoma cells and cooperate with other immune cells to develop adaptive immune responses. They directly mediate melanoma cell lysis by releasing perforin and granzyme B [24,125]. Murine studies proved that NK cell depletion is associated with decreased survival in melanomas [126]. The function of NK cells may be altered by multiple soluble factors produced in the melanoma microenvironment by CAFs, macrophages, dendritic cells, and Tregs [127,128]. The suppressive effects of other cells on NK cells increase as melanoma progresses, gradually exhausting NK cells [129]. NK cells lose their ability to respond to melanoma cells, in part due to diminished expression of activating receptors, such as the Natural Killer Group 2 Member D receptor (NKG2D) [130]. By upregulating NKG2D ligand expression, chemotherapy agents such as dacarbazine and cisplatin may enhance NK cytotoxicity [24,131]. Furthermore, the acidic environment resulting from melanoma cells producing excessive lactate during glycolysis negatively affected the number and activity of NK cells [132]. Importantly, NK cells can be restored by neutralizing the acidic microenvironment [133].

*NK cells provide a critical first line of defense against MHC-deficient melanoma cells. However, tumor-derived TGF-β and metabolic stressors often lead to NK cell exhaustion, making their “re-activation” a promising frontier for cellular therapies. Conversely, a high Treg-to-CD8 ratio is a major barrier to immune checkpoint inhibitors (ICIs).*

Dendritic cells (DCs) typically present antigens to T cells, triggering the adaptive cytotoxic activity against melanoma cells. DCs become activated via Toll-like receptors (TLRs) and subsequently produce proinflammatory cytokines and interferons [24,134]. However, DCs may express multiple TLR subtypes. For instance, binding of TLR2 ligands produced by melanoma cells to TLR2 impairs DC activity [135]. Furthermore, as melanoma progresses, VEGF and other soluble factors secreted by microenvironmental components hinder DC maturation. Malfunctioning DCs promote melanoma progression through various mechanisms that are yet to be entirely elucidated. These cells may produce high levels of adenosine, which alters cross-priming presentation [134]. Additionally, DCs may support melanoma growth by expressing immune checkpoint receptors [134,136].

Langerhans cells (LCs) are the only DC subset in the epidermis and the dominant population in melanoma sentinel lymph nodes (SLN). Their function is suppressed in the SLN by tumor-derived IL-10 and TGF-β, which downregulate costimulatory and maturation markers. Consequently, LCs remain immature, promoting tolerance and early metastasis. A possible therapeutic target is IDO1, as its expression in CD83^−^ LCs further increases immunosuppression [137].

According to mouse melanoma models, plasmacytoid dendritic cells (pDCs) can exert cytotoxicity via TRAIL and granzyme B. However, their presence often correlates with poor prognosis, as pDCs frequently acquire a tolerogenic phenotype. Tolerogenic pDCs promote Treg growth and inhibit effector responses by expressing ICOS-L, IDO, and PD-L1. Granzyme B’s dual function and therapeutic potential are highlighted by its ability to both induce tumor cell death and suppress T-cell activation when released by pDCs [15,138].

*Melanoma induces a “tolerogenic” state in DCs, impairing their ability to prime T cells. Reversing this dysfunction through TLR agonists or IDO inhibitors is a primary experimental strategy to restore the efficacy of the adaptive immune response. Furthermore, the maturation state of LCs in SLN is an emerging prognostic indicator for the risk of early systemic spread.*

Myeloid-Derived Suppressor Cells (MDSCs) are immature myeloid cells that represent the precursors of DCs, macrophages, and granulocytes [139]. They can be divided into two major categories based on their phenotypic and morphological resemblance to either monocytes or granulocytes: monocytic (M-MDSCs) and polymorphonuclear (PMN-MDSCs) [140]. Overall, MDSCs promote melanoma growth due to their immunosuppressive activity [141]. MDSCs enhance the metastatic potential of melanoma by expressing high levels of Wnt5A [142]. Melanoma-derived microRNAs can induce the conversion of mature myeloid cells into MDSCs [143,144]. M-MDSCs are present in the melanoma microenvironment and promote immunosuppression [145,146]. In melanoma, C-C chemokine receptor type 5 (CCR5) ligands stimulate migration and expansion of M-MDSCs [128,147]. In this respect, melanoma growth can be inhibited by administration of CCR5-Ig fusion protein in murine models [147]. Similarly, CXC chemokines stimulate PMN-MDSC recruitment, and deletion of chemokine receptor 2 (CXCR2) reduces their accumulation and melanoma growth in mice. [148].

Finally, high levels of MDSCs in the TME have been linked to resistance to both CTLA-4 blockade and BRAF inhibitors in melanoma. In patients treated with ipilimumab, elevated circulating MDSCs correlated with poor response [149]. Similarly, in BRAF inhibitor-resistant mouse models, reactivation of MAPK signaling promoted cytokine release (e.g., CCL2), driving MDSC accumulation and immune suppression [150].

*MDSCs are traditionally characterized as potent inhibitors of T-cell activity; however, they exhibit significant functional plasticity. While the majority of tumor-infiltrating MDSCs drive immune evasion through L-arginine depletion and oxidative stress, emerging evidence suggests that certain subsets can be “re-programmed” toward an anti-tumor phenotype. Recognizing this duality is essential for clinical translation, as the goal shifts from simple depletion to the therapeutic modulation of MDSC polarization to restore an immuno-permissive TME.*

Mast cells are innate immune cells resident in the dermis that play a multifaceted, yet still not fully elucidated, role in the CM microenvironment. An immunohistochemical study demonstrated that mast cell serine proteases, tryptase and chymase, are reduced in invasive melanomas compared with benign, dysplastic, and in situ lesions. Low tryptase levels are associated with poorer survival and advanced tumor stage, while low chymase correlates with microsatellites [151]. Similarly, another immunohistochemical study demonstrated that more locally advanced melanomas display significantly lower numbers of intratumoral and peritumoral mast cells [152]. In contrast, other immunohistochemical studies found significantly higher numbers of mast cells in more advanced melanomas and in patients who developed metastases and died during follow-up [153,154]. This increased density in and around primary melanoma lesions may contribute to both tumor progression and immune modulation. Mast cells release a wide array of mediators, including cytokines (IL-6, TNF-α), chemokines, and VEGF, which collectively promote angiogenesis, ECM remodeling, and melanoma cell invasion [155]. Recent RNA-seq analyses of mast cells purified from melanoma biopsies reveal a distinct transcriptomic profile, including strong upregulation of the complement component C3. Increased C3^+^ mast cells correlate with advanced melanoma stages and poor survival. In vitro, melanoma-derived cytokines can induce these transcriptional changes, highlighting how tumor-secreted factors drive a functional mast cell phenotype switch that negatively impacts prognosis [156]. Melanoma cells can also suppress mast cells’ function via a melanin-dependent mechanism, which may promote an immunosuppressive pro-tumoral microenvironment [157]. Having considered all the evidence, mast cells appear to play a dual role in CM. On one hand, studies showing that higher densities of tryptase- and chymase-positive mast cells correlate with better survival suggest a protective, anti-tumorigenic role in certain contexts. Conversely, they can promote tumor progression by secreting pro-angiogenic factors, proteases, and immunomodulatory mediators that shape a tumor-supportive microenvironment. These findings highlight that the impact of mast cells in melanoma is context-dependent, influenced by their localization, activation state, and interactions with tumor and immune cells.

*Mast cells play an ambivalent role in the melanoma TME. While they can support anti-tumor immunity through TNF-α and protease release in early-stage lesions, they more commonly promote tumor progression in advanced disease by driving angiogenesis (via VEGF and Heparin) and facilitating immune escape. This dualistic role makes them a controversial biomarker, suggesting that their clinical impact is strictly context-dependent rather than universally pro- or anti-tumoral.*

#### 4.1.4. Keratinocytes

Keratinocytes play a significant role in preventing UV-induced mutations in melanocytes, and the melanin transferred from melanocytes acts as a shield against UV radiation. Furthermore, keratinocytes produce various growth factors and cytokines that modulate the proliferation, adhesion, migration, and differentiation of melanocytes. Nevertheless, they can also promote melanoma development and progression [158]. During the earliest stages of melanoma genesis, keratinocytes exposed to UV radiation undergo stress responses that include the secretion of proinflammatory cytokines and growth factors that promote melanocyte proliferation and survival [159]. These signals can cooperate with acquired oncogenic mutations such as BRAF V600E to enable melanocytes to evade oncogene-induced senescence, a key barrier to early tumor formation [160]. UV-damaged keratinocytes also release reactive oxygen species, prostaglandins, and matrix metalloproteinases, which generate a mutagenic and inflammatory epidermal microenvironment that promotes melanocyte genomic instability and migration [159,160]. In normal skin, keratinocytes tightly regulate melanocyte proliferation and differentiation via adhesion molecules such as E-cadherin. The loss of E-cadherin in melanoma cells disrupts this control and facilitates invasion [161]. Melanoma cells also reprogram adjacent keratinocytes, altering their differentiation pattern and cytokine secretion profiles [162]. Recent evidence shows that melanoma-derived factors suppress desmoglein 1 expression, a cadherin-type cell adhesion molecule, in keratinocytes, triggering the release of pro-migratory chemokines which enhance melanoma motility [163]. Deficiency of desmoglein 1 may also promote pagetoid spread, an early event in melanoma development [164]. In turn, keratinocytes contribute to a tumor-supportive milieu by secreting cytokines and growth factors including IL-1, IL-6, IL-8, TNF-α, VEGF, and FGF-2, which promote melanoma proliferation, angiogenesis, and epithelial-to-mesenchymal-like transition [159,162,165]. Moreover, keratinocyte-secreted thymic stromal lymphopoietin (TSLP) shapes an immunosuppressive microenvironment by expanding Tregs and skewing DC polarization, thereby facilitating melanoma growth and metastasis [166]. Furthermore, keratinocytes also drive ECM remodelling. For instance, keratinocyte-derived ECM proteins such as laminin-332 further enhance melanoma adhesion and invasion [167]. Keratinocytes exposed to melanoma secretome up-regulate matrix metalloproteinases, thus remodeling the extracellular matrix and facilitating invasion [168].

Collectively, these alterations illustrate how keratinocytes actively drive melanoma initiation and progression through loss of adhesion control, paracrine activation, and creation of a pro-tumorigenic inflammatory niche.

Keratinocyte–melanoma interactions are emerging as a possible therapeutic interface in the epidermal tumor microenvironment. While there is presently no direct targeting of keratinocytes in melanoma treatment, there are several approaches that try to disrupt keratinocyte-derived inflammatory signaling that encourages melanocyte transition and tumor growth. Melanocyte proliferation and early melanoma initiation may be supported by an inflammatory epidermal niche induced by ultraviolet irradiation, especially UVB, which causes keratinocytes to secrete pro-inflammatory cytokines [169]. Thus, the rationale for targeting this pathway in inflammation-driven malignancies is supported by the fact that IL-1 signaling has been implicated in melanoma progression and stromal-mediated resistance to MAPK pathway inhibition [170,171].

*Keratinocytes are critical gatekeepers whose role shifts from protective to pro-tumorigenic during melanomagenesis. By losing E-cadherin-mediated control and secreting pro-migratory factors, they create a mutagenic and inflammatory epidermal microenvironment that facilitates early invasion and shields melanoma cells from immune recognition.*

**Figure 1:**
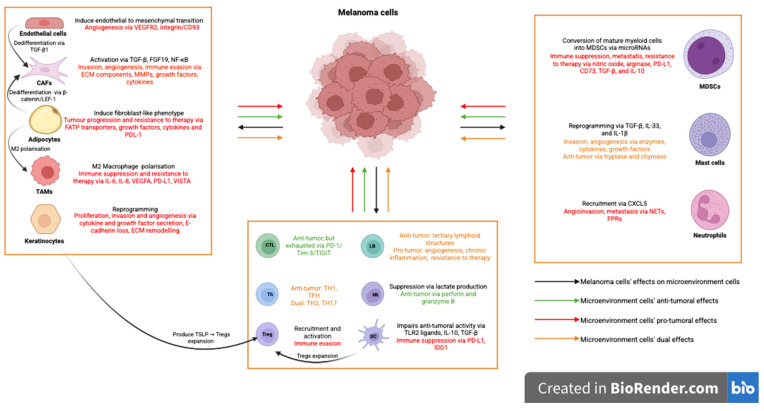
Interactions between melanoma and microenvironment cells.

The complex interactions between melanoma cells and the microenvironment are highlighted in Figure 1.

### 4.2. Non-Cellular Components

Through cell-to-matrix interactions and ECM remodeling, bidirectional crosstalk between resident cells and the ECM dynamically shapes tissues. Tumors use ECM remodeling to establish a background conducive to tumorigenesis and metastasis [172,173]. Fibrillar and structural proteins, such as collagen types I, III, VII, XV, and XVIII, laminin, tenascin-C, and fibronectin, as well as hydrated gel-forming macromolecules, such as hyaluronan and proteoglycans, are included in the canonical ECM. Additionally, integrins, which are responsible for adhesion signaling, and several other components are also included in the vast milieu of the ECM [174]. Each ECM component controls cellular processes and provides structural support to surrounding cells.

#### 4.2.1. Extracellular Matrix: Composition, Remodeling, Role of Matrix Metalloproteinases, Adhesion Molecules

As melanoma cells proliferate and migrate rapidly into the dermis, the basement membrane components eventually diminish. The aberrant distribution of type IV collagen will mainly affect the regional architecture. Thus, the degree of invasion and distant metastasis can be influenced by the loss of the typical boundary characteristics between epithelial and connective tissues [174,175].

During melanoma progression, the expression of ECM proteins, including fibronectin and tenascin, increases [176]. The altered molecular profile of these adhesion proteins, corroborated by the observation that melanomas exhibit somewhat thicker collagen bundles than melanocytic nevi but fewer altogether, remodels the ECM’s three-dimensional network to support melanoma growth and metastasis [177]. Furthermore, histopathological analysis reveals that melanoma nests are closely encircled by dermal collagens I, III, and VI with notable changes in proteoglycan distribution [174]. Consequently, the stroma surrounding melanoma cells shows high Versican expression [178].

Indicators of advanced melanoma include adhesion molecules, which prevent melanoma cells from typically undergoing epithelial-to-mesenchymal changes and instead promote mesenchymal-to-epithelial transition states [179].

Multiple variables in a melanoma cell’s environment can alter its morphology. These can include soluble factors (such as TGFb and IFNg), insufficient nutrients or oxygen, or oncogenic BRAF-targeting treatment [180]. In addition, it seems that tumor stiffness is caused by increased deposition and modification of the ECM by both stromal and cancer cell-derived components [181]. It is well known that poor clinical outcomes and increased tumor aggressiveness are associated with a stiff and fibrotic TME [180]. In patients with relapsed CM following the failure of BRAF inhibitor treatment, recent observations have demonstrated that elevated collagen expression is associated with poor patient survival and an increase in fibrous ECM [182]. Also, by changing immune cell infiltration and triggering myofibroblastic-cancer-associated fibroblasts activation, increasing ECM density and mechanical characteristics may reduce treatment effectiveness [183]. Mechanical cues modulate ECM stiffness to affect melanoma cell phenotype [180]. By interacting with specific components of the mechanotransduction pathway, members of the integrin and collagen receptor-discoidin domain tyrosine kinase receptors (DDR) can detect the physical properties of the ECM [184]. Melanoma progression is accompanied by a rise in DDR1 and DDR2 levels, which are selectively enriched in dedifferentiated melanoma cell subpopulations [180,184]. Additionally, the proliferation, invasion, and survival of human melanoma cell lines are reduced by targeting DDR1, whereas the metastatic potential of murine melanoma cell lines is reduced by depleting DDR2 [184,185,186].

Migration, adhesion, and invasion of melanoma cells are all impacted by the TME’s acidity. By altering the interaction between cells and ECM, Krähling et al. showed that extracellular pH influences the migration of human melanoma cells [187]. Thus, when the contact is too strong at acidic pH or too weak at alkaline pH, migration is hindered [188]. Certain molecules, including integrins α2β1, α5β1, and αVβ3, have a pH-dependent behavior in the context of melanoma [187,189]. In addition, melanoma cells can shift from an epithelial to a mesenchymal architecture when exposed to an acidic environment [188].

Proteolysis of the ECM and degradation of different ECM constituents are essential functions of MMPs, matrixins, or zinc- and calcium-dependent endopeptidases [190]. The immune system (neutrophils, macrophages, DCs), tumor cells, keratinocytes and fibroblasts intrinsically produce MMPs [191]. While protein breakdown alters the immune milieu, MMPs are crucial for tissue remodeling, angiogenesis, tissue repair, local invasion, and metastasis [192,193]. In a melanoma model, angiogenesis and tumor growth were promoted by enhanced production of prostaglandin E2 and MMP-9 by TAMs [194]. By imitating the actions of endothelial cells and generating microvascular channels, the most aggressive forms of melanoma can achieve vasculogenic mimicry. Proteinases and angiogenic factors facilitate this process [195].

The activity of MMPs is regulated by endogenous tissue inhibitors of metalloproteinases (TIMPs) and synthetic MMP inhibitors (MMPIs), both of which are currently being assessed as prospective cancer therapies [174]. Due to severe side effects and a lack of a statistically meaningful decrease in tumor development, broad-spectrum MMPIs were removed from phase III clinical trials despite promising preclinical findings [195,196,197].

The ECM is an active driver of melanoma biology—modulating transformation, invasion, immune evasion, and therapy resistance through mechanical forces, molecular signals, and structural remodeling.

#### 4.2.2. Cytokines and Growth Factors

Melanoma cells and parts of the microenvironment can interact bidirectionally, ultimately releasing several soluble substances that contribute to local inflammation [198]. Cytokines and growth factors use autocrine and paracrine mechanisms to promote tumor development and invasion [199].

Accumulating evidence suggests that melanoma patients experience local and systemic immunosuppression due to the continuous production of inflammatory molecules. This, in turn, is linked to tumor development and metastasis [200,201]. The scientific community that studies melanomas has recently emphasized measuring serum cytokines, chemokines, and growth factors as potential biomarkers [200,201,202,203,204].

In 2021, Yoel Genaro Montoyo-Pujol et al. observed a reproducible molecular signature consisting of high/intermediate levels of VEGFA, IL6, MCP-1, IL-8, SDF-1, HGF, MIP-1β, GRO-α, and LIF in pre-metastatic and metastatic melanoma-generated cultures [198]. The authors conclude that melanoma cells generate BDNF, FGF-2, and NGF-β in addition to VEGFA and that these molecules may be crucial for the melanoma spreading process.

TGF-β and platelet-derived growth factor (PDGF) are two tumor-derived growth factors that can convert various stromal cells in the TME into cancer-associated stromal cells [205,206]. Fibroblasts undergo a transformation into CAFs when they interact with tumor cells via the production of tumor-cell-derived TGF-β [207]. The activation of CAFs produces additional cytokines and chemokines. This leads to a detrimental loop with tumor progression and drug resistance [207]. An overview of the most significant cytokines and growth factors expressed by melanoma is illustrated in Table 1.

#### 4.2.3. Hypoxic Environment

Melanoma aggressiveness is driven by hypoxia, which also supports tumor growth and spread via HIF-mediated signaling, altering metabolism, angiogenesis, immune evasion, senescence, treatment resistance, apoptosis suppression, and invasiveness.

**Figure 2:**
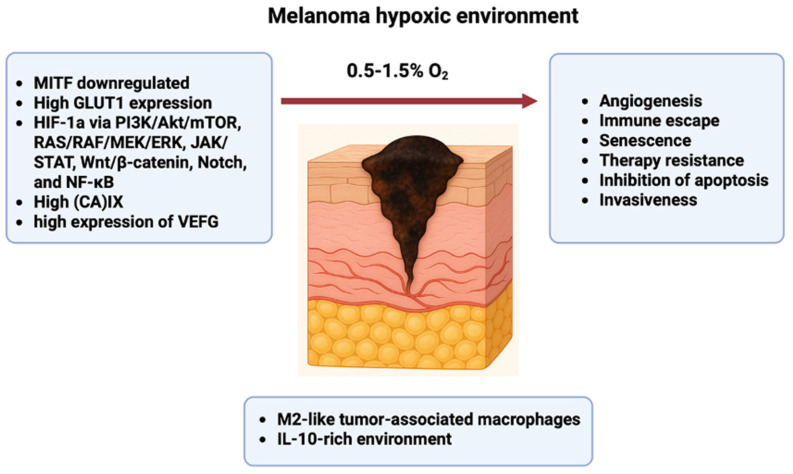
Hypoxic microenvironment in CM and its biological consequences. Melanoma tumors often develop regions of hypoxia (0.5–1.5% O_2_), leading to significant molecular and cellular adaptations. Hypoxic signaling downregulates the melanocytic lineage factor MITF and increases GLUT1 expression, indicating metabolic reprogramming. Activation of HIF-1α stabilizes multiple oncogenic pathways (e.g., PI3K/Akt/mTOR, RAS/RAF/MEK/ERK, JAK/STAT, Wnt/β-catenin, Notch, and NF-κB), upregulates carbonic anhydrase IX (CAIX), and promotes VEGF expression, contributing to tumor survival. The hypoxic milieu also supports immunosuppressive M2-like tumor-associated macrophages and an IL-10-rich environment. Collectively, these changes enhance angiogenesis, immune evasion, senescence, therapy resistance, apoptosis inhibition, and invasiveness, supporting tumor progression and metastasis.

Regions of hypoxia and anoxia (where oxygen level varies from 0.5–1.5% O_2_) brought on by a disparity in oxygen consumption and supply occur in melanoma tumors, much as in other malignancies [229,230,231]. Extensive research has shown that tumor hypoxia may cause gene amplification, potentially leading to treatment resistance [232] (Figure 2).

Microphthalmia-associated transcription factor (MITF), the primary regulator of melanocyte development, is downregulated in response to hypoxia [231]. Highly used for detecting melanocytic lesions, the melanoma antigen recognized by T cells 1 (MLANA) is also implicated in melanosome formation [233,234]. High GLUT1 expression in immunohistochemical stainings of melanoma indicates hypoxic areas that negatively correlate with MITF and MLANA. Thus, downregulation of melanocytic markers in hypoxic microenvironments indicates cell dedifferentiation, particularly in invasive phenotypic cells [231].

Hypoxia-inducible factor (HIF) is activated by tumor hypoxia, which regulates gene expression in protein synthesis, pH regulation, DNA replication, and metabolic pathways [235]. Under normal settings, HIF1α undergoes hydroxylation, eventually leading to its proteasomal degradation [236]. Conversely, hydroxylation of HIF1α is not possible when oxygen levels are inadequate. The subsequent accumulation and movement into the nucleus to form a dimer with HIF1β will eventually control the expression of several genes [236]. Carbonic anhydrase (CA)IX is encoded by one of these genes and is a significant player in controlling pH, adhesion, migration, and tumor cell survival [237]. Melanoma patients with an elevated level of this enzyme have a poorer prognosis and are more likely to develop metastases [188].

By controlling the expression of genes related to angiogenesis, metabolism, cell proliferation, metastasis, and other biological processes, hypoxia activates HIF-1α, promoting tumor development [238]. In addition, via controlling the expression of insulin-like growth factor 2 (IGF2), cellular myelocytomatosis (C-MYC), and other cell cycle and death pathway regulators, HIF-1α influences cell proliferation and death [239].

Signaling pathways like Ras/MAPK, PI3K/AKT, or NF-κB, which are often upregulated in cancer cells, could stimulate HIF-1α in a hypoxia-independent manner in response to growth factors and cytokines [240,241]. Hao et al. demonstrated that overexpression of HIF-2α induced stemness in melanoma cells by inhibiting p21. In addition, the authors observed a positive correlation between poor prognosis and high expression of VEGF and HIF-2α in samples isolated from nodular CM [242].

Therefore, the entire construct, composed of ECM and cells, works together to affect the dynamics of tumor mass spatiotemporal growth by promoting hypoxia and an acidic environment. This idea is supported by the fact that melanoma tumor cells generate in response to hypoxia the damage-associated molecular pattern High-Mobility Group Box1 protein (HMGB1), which favors the M2-like TAMs and an IL-10-rich environment within the tumor [243].

## 5. Therapeutic Implications and Future Perspectives

Significant therapeutic challenges persist in melanoma management. The development of resistance to systemic therapies, the poor prognosis associated with brain metastases, and the limited efficacy of current treatments for rare melanoma subtypes underscore the necessity for continued innovation [244,245,246]. Emerging diagnostic and prognostic biomarkers, including novel immunohistochemical markers, gene mutation analyses, microRNAs, and exosome-derived proteins, hold promise for enhancing early detection, risk stratification, and personalized therapeutic strategies [247,248]. The integration of multi-omics data, advanced preclinical models, and patient-centered research approaches is anticipated to drive further progress in understanding melanoma biology and overcoming therapeutic resistance [249,250].

The translation of the melanoma TME from a biological concept into clinical practice requires a clear distinction between validated biomarkers, current standards of care, and experimental frontiers.

### 5.1. Prognostic Landscapes: Established vs. Controversial Markers

While many TME components influence progression, their clinical utility as biomarkers varies significantly:

1. **Established prognostic markers:** The presence of CD8^+^ cytotoxic T lymphocytes (CTLs) and B-cell signatures—specifically within Tertiary Lymphoid Structures (TLS)—is currently the most robust indicator of positive clinical outcomes and response to immunotherapy. Multiparameter immune and molecular profiling stratifies melanoma patients, enabling personalized TME-directed therapies. Circulating tumor DNA (ctDNA) levels and TME transcriptomic signatures predict responses and resistance mechanisms [251,252,253,254]. Integrating biomarker data guides rational therapeutic combinations and sequences, optimizing efficacy and minimizing toxicity.
2. **Controversial/context-dependent markers:** As detailed in Section 4, while high densities of TAMs, CAFs, and MDSCs generally correlate with poor prognosis, their role can be ambivalent. For example, total macrophage density sometimes shows no association with survival unless polarized to the M2 phenotype [8,73]. Similarly, the role of mast cells remains a subject of ongoing research and lacks a definitive consensus for routine clinical use.

### 5.2. Therapeutic Targeting: Current Standards vs. Experimental Frontiers

Therapeutic innovation is currently divided between optimizing established checkpoints and targeting the supportive stroma:

1. **Targetable today (standard of care):** Actionable clinical targets are dominated by the PD-1/CTLA-4 axes. New clinical trials are actively evaluating novel immune modulators such as checkpoint molecules Lymphocyte activation gene 3 (LAG-3), T-cell immunoglobulin and mucin-domain containing-3 (TIM-3), and TIGIT, which are upregulated on exhausted T cells within melanoma TME [255,256,257,258]. Notably, the recent integration of LAG-3 blockade has set a new standard, with dual LAG-3/PD-1 inhibition doubling progression-free survival (PFS) compared to monotherapy [259,260]. Bispecific antibodies targeting these receptors amplify TCR signaling, increasing CTL activation while preserving tolerability [261,262]. Oncolytic virotherapy (T-VEC) also remains a standard local intervention to trigger immunogenic cell death [263]. Oncolytic virotherapy employs viral vectors to trigger immunogenic tumor cell death and potentiate immune recognition [264,265]. Oncolytic viruses combined with checkpoint inhibitors increase T cell infiltration and tumor regression [266,267,268]. Talimogene laherparepvec (T-VEC) combined with pembrolizumab achieved a 39% important objective response rate (ORR), outperforming pembrolizumab monotherapy [263,269].
2. **Experimental frontiers (clinical trials):** Several clinical studies have investigated the potential therapeutic roles of melanoma microenvironmental cellular components. For instance, Colony-stimulating factor 1 receptor (CSF1R) blockade reprograms macrophage phenotypes, enhancing CTL activity. In this context, Pexidartinib, a CSF1R inhibitor, has proven constructive interaction with anti-PD-1 antibodies, improving progression-free survival (PFS) in metastatic melanoma [270,271]. Phase II trials confirmed enhanced tumor regression and immune activation with combination regimens [272]. Next-generation strategies are also focusing on the “non-immune” TME. CAFs produce C-X-C Motif Chemokine Ligand 12 (CXCL12), which facilitates T cell exclusion and immunosuppression [273]. C-X-C Motif Chemokine Ligand 4 (CXCR4) antagonism, via AMD3100, disrupts this axis, allowing T cell tumor infiltration [274,275,276]. A clinical trial combining AMD3100 with pembrolizumab yielded an important objective response in PD-1 refractory melanoma [277]. Matrix metalloproteinase inhibitors such as Marimastat modulate ECM remodeling to decrease invasiveness and improve immune cell access [172]. Epigenetic modifiers, including DNMT and HDAC inhibitors, reverse tumor antigen silencing by increasing Major Histocompatibility Complex (MHC) expression and neoantigen presentation [278,279]. Notably, Entinostat combined with anti-PD-1 has yielded significant modifications of ORR in resistant models, with clinical trials reporting promising responses and manageable toxicity [280,281,282,283,284]. Regarding soluble factors, TGF-β signaling blockade with galunisertib reduced Treg recruitment and fibrosis, thereby increasing CD8^+^ T cell infiltration [285,286,287]. IL-10 receptor blockade activated DCs, augmenting antigen presentation and T cell priming [288,289,290,291]. These cytokine neutralizations, combined with checkpoint inhibitors, prove synergistic tumor control, supporting further clinical investigation [292].

### 5.3. Evidence Levels: Preclinical vs. Human Outcome Data

A significant challenge for clinicians is the “translational gap” between laboratory models and patient outcomes:

1. **Preclinical/early-phase data:** Many metabolic and physical interventions remain largely in the preclinical or Phase I stage. Metabolic reprogramming in melanoma generates elevated lactate, which suppresses CTL function and promotes TAM differentiation [293]. Lactate dehydrogenase A (LDHA) inhibitors reduce lactate production, thereby restoring immune activity [294,295]. The glycolytic inhibitor 2-deoxy-D-glucose (2-DG) synergizes with PD-1 blockade, increasing CD8^+^ T cell tumor infiltration [296,297,298]. Early-phase clinical studies indicate LDHA inhibition combined with checkpoint blockade is well-tolerated and promotes tumor regression [299,300]. Combination therapies that target hypoxia pathways modulate the immune landscape and improve clinical outcomes. Hypoxia within melanoma TME upregulates HIF-1α, increasing VEGF and PD-L1 expression [188,301]. Evofosfamide, a hypoxia-activated molecule, selectively kills hypoxic melanoma cells, thereby enhancing the efficacy of PD-1 blockade and improving the response rate in murine models [302,303].
2. **Biomaterial-based systems** deliver immunomodulators locally within melanoma TME. In this respect, early phase data have also shown that STING agonist-laden hydrogels enhanced DC maturation and CD8^+^ T cell recruitment, doubling survival in preclinical melanoma models [304,305,306]. Extracellular vesicles (EVs) mediate intercellular communication within melanoma TME by transferring immunosuppressive agents [307,308,309]. Suppressing EV biogenesis through a neutral sphingomyelinase (SMase) inhibitor—the GW4869—reduces metastasis and enhances T cell activation in murine models [310,311,312]. EV-based therapeutic platforms are under development for the targeted delivery of immunomodulators [313,314,315]. Nanoparticle-mediated siRNA targeting PD-L1 or IDO improved tumor-specific immune activation and reduced systemic exposure [316,317,318].
3. **Human-validated data:** Strategies such as vasculature normalization with Bevacizumab are supported by high-level clinical evidence. Bevacizumab normalized vasculature and synergized with ipilimumab, extending median overall survival by six months compared to ipilimumab monotherapy [319,320,321]. Similarly, dual checkpoint blockade with LAG-3 and PD-1 prolongs PFS and induces durable responses [259,322,323]. The most promising clinical results involve multi-target strategies. Trials combining CSF1R and CXCR4 inhibitors with PD-1 blockade have yielded ORRs exceeding 40% in refractory populations [324,325,326,327]. Furthermore, combination therapies targeting different TME components with systemic checkpoint inhibitors continue to produce enhanced antitumor effects across diverse patient cohorts [15,328,329,330].

To facilitate the translational interpretation of the melanoma tumor microenvironment, Table 2 provides an integrative overview linking the principal microenvironmental components discussed in this review with their prognostic relevance and potential therapeutic implications.

## 6. Conclusions

The melanoma microenvironment is integral to tumor progression, immune escape, and therapy resistance. The stromal cells and components of the ECM modulate tumor plasticity, providing physical niches for metastatic spread and facilitating adaptive resistance through various soluble factors such as cytokines, chemokines, and growth factors. The immune cell infiltration patterns within the microenvironment critically influence immunotherapy response.

Recent advancements in melanoma treatment have profoundly changed the patient management strategies. The novel agents target both tumor cells and the melanoma microenvironment, leading to improved outcomes. In parallel, ongoing research into the interactive niche surrounding melanoma highlights new therapeutic opportunities involving immune modulation, stromal signaling, and metabolic reprogramming. Emerging systemic treatments for melanoma focus on next-generation immunotherapies and molecularly targeted approaches. Thus, intriguing agents such as immune checkpoint inhibitors targeting LAG-3, TIGIT, and TIM-3, either alone or in synergistic combinations with established PD-1/PD-L1 and CTLA-4 inhibitors, have demonstrated enhanced antitumor immune activation and improved survival in advanced melanoma patients. However, adoptive cell therapies, particularly tumor-infiltrating lymphocyte (TIL) infusion, CAR-T cells adapted for melanoma, or dendritic cell vaccines, are showing promising results in selected populations by counteracting immune evasion mechanisms.

Metabolic rewiring, including hypoxia-driven lactate production and lipid metabolic changes, fosters immunosuppression and therapy resistance, suggesting working therapeutic alternatives for metabolic inhibitors as adjuvant therapies in melanoma. The convergence of refined immunotherapeutic modalities and microenvironmental modulation offers attractive prospects for durable melanoma control, particularly in metastatic and refractory disease settings. Robust preclinical and early clinical data indicate that dual or triple blockade strategies addressing both tumor-intrinsic signaling and key microenvironmental suppressors may overcome acquired resistance that limits monotherapies.

Despite significant progress, several knowledge gaps hinder the full clinical translation of TME-directed therapies. First, the temporal evolution of the TME—how it changes from primary lesion to metastatic site—remains poorly understood. Second, unresolved controversies persist regarding the dualistic nature of certain cells, such as mast cells and Th17 lymphocytes, which exhibit both pro- and anti-tumoral functions depending on the cytokine milieu. Finally, a major translation barrier exists in the lack of standardized, high-throughput assays to quantify TME “states” in routine clinical practice, a necessity for truly personalized immunotherapy.

Continued investigation into the dynamic interplay between melanoma cells and their niche is likely to yield novel druggable targets and combinatorial strategies, boosting the prospect of long-term remission or cure for greater numbers of patients. Personalized approaches based on microenvironmental profiling, including spatial transcriptomics and multiplex immune imaging, promise to optimize patient selection for emerging treatments and minimize adverse effects. These scientific developments underscore how an integrative approach—encompassing both direct tumor targeting and rational microenvironmental intervention—represents the most promising horizon for the future of melanoma therapy.

## Figures and Tables

**Table 1:** Key cytokines and growth factors generated within the melanoma TME.

Interleukin	Roles	Ref
**IL-1α**	tumor-specific and Th1 immunity are both suppressed by IL-1-mediated signaling, which in turn increases immunotherapy resistance	S. Singh et al., 2021 [208]
**IL-4**	antitumor effect via increased activating NKG2D receptor expression and IL-4-induced NK cell cytotoxicity	Vuletić et al., 2020 [209]
**IL-6**	elevated levels—correlate with advanced disease and therapy resistance	Tanaka et al., 2014 [210] K. P. Singh et al., 2024 [211]
**IL-8**	binds to CXCR-1 and CXCR-2: melanoma proliferation and metastasishighly expressed by anti-apoptotic, aggressive tumor cells	Filimon et al., 2022 [212] Elias et al., 2010 [199]
**IL-10**	tumor cell IL-10 mRNA is associated with increased Clark’s level, indicating vertical tumor progression	Itakura et al., 2011 [213]
**IL-13**	IL13Rα2 is a potential therapeutic target as it promotes tumorigenesis via angiogenesis	Okamoto et al., 2019 [214]
**IL-15**	the TME may be sensitized to immune checkpoint therapy through gene-based IL-15 delivery	Awad et al., 2023 [215]
**IL-17**	potential biomarker for determining the efficacy of dual-immune checkpoint inhibition therapy	Váraljai et al., 2023 [216]
**IL-18**	IL18 expression predicts melanoma survival and correlates with CD8+ T and NK cell infiltration	Gil & Kim, 2019 [217]
**OPN**	increased Breslow thickness and mitotic index were associated with higher osteopontin levels	Levati et al., 2024 [218]
**TNF-α**	TNF-α secretion and MMP-2 enzymatic activity cooperate to define the aggressive phenotype of melanoma cells	Rossi et al., 2018 [219]
**IFN-α**	IFN alpha-2b increases high-risk resected melanoma patients’ overall survival and relapse-free period	Kirkwood et al., 2023 [220]
**GM-CSF**	the effects of antimelanoma peptide or allogeneic tumor cell vaccines are not improved by systemic administration of GM-CSF	O Dillman, 2020 [221]
**Growth** **Factors**	**Roles**	**Ref**
**TGF-β1**	low and high levels of TGFβ1 alone cannot cause cell death, but when the MAPK pathway is simultaneously inhibited, high levels of TGFβ1 have a potent pro-apoptotic impact	Loos et al., 2024 [222]
**VEGFA**	VEGF/VEGFR axis impacts melanoma cell growth, proliferation, migration, metastasis, survival, and acquired therapeutic resistance	Malekan et al., 2024 [223]
**PDGF**	PDGF activates downstream signaling pathways including MAPK/ERK and PI3K/Akt to promote melanoma cell growth	Cazzato et al., 2024 [224]
**IGF-1**	melanoma progression is independently influenced by serum IGF1 levels	Castillo-Ferrer et al., 2024 [225]
**PlGF**	melanoma patients had a 20-fold rise in plasma PlGF levels during bevacizumab-containing treatment reduced VEGFA and increased PlGF	Pagani et al., 2016 [226]
**FGF**	a new selective FGFR inhibitor—CPL304110, is highly successful in halting the growth of primary A375 and metastatic RPMI7951 melanoma cell lines	Piotrowska et al., 20223 [38]
**EGF**	melanoma EGFR expression was shown to affect sentinel lymph node metastatic invasion and tumor progression	Pastwińska et al., 2022 [227] Boone et al., 2011 [228]

**Table 2:** Translational relevance of melanoma tumor microenvironment components: prognostic significance and therapeutic implications.

TME Component	Key Markers	Pro/Anti-Tumor Role	Clinical Status/Candidate Therapy
CAFs	α-SMA, FAP	Pro-tumor—ECM remodeling/resistance	Clinical trial: CXCL12 inhibition
Adipocytes	FATP, PD-L1, IL-6	Pro-tumor via lipid transfer & immune escape	Experimental: FATP inhibitors/metabolic modulators
Keratinocytes	E-cadherin, IL-1, MMP-19	Pro-tumor (via loss of adhesion & paracrine signaling)	Experimental: MMP inhibitors/TSLP blockers
Endothelial Cells	CD31, CD93	Pro-tumor—Angiogenesis	Targetable: VEGF/CD93 blockers:
Neutrophils	CD66b, NLR	Pro-tumor—NETs release	Prognostic: blood-based biomarker
M2 Macrophages	CD163, CD206	Pro-tumor Immunosuppressive	Clinical trial: CSF1R inhibitors
TFH cells	CXCR5^+^, PD-1	Anti-tumor	Prognostic: Supports TLS formation, correlates with survival
Treg cells	FoxP3^+^, CD25^+^	Pro-tumor Immunosuppression	Standard: anti-CTLA-4 depletion
CD8^+^ T-cells	CD8^+^, PD-1, LAG-3	Anti-tumor/Target	Standard: ICIs (PD-1/CTLA-4/LAG-3)
B-cells	CD20, CXCL13	Anti-tumor	Prognostic: Predicts ICI response
NK cells	CD56^+^, CD16^+^	Anti-tumor/Cytotoxicity	Experimental: NK-cell engagers/Cytokines
Dendritic Cells	CD83^+^, HLA^-^DR	Anti-tumor—Antigen presentation	Experimental: TLR agonists/STING agonists
MDSCs	CD11b, CD33	Ambivalent	Insufficient data
Mast Cells	Tryptase, CD117	Ambivalent	Controversial: Research focus only
ECM	FAP, MMPs	Pro-tumor—Barrier/ Invasion	Clinical trial: Marimastat/CXCR4 inhibitors
Metabolic reprogramming	Lactate, LDHA	Pro-tumor Immunospression	Preclinical: LDHA/2-DG inhibitors
Hypoxia	HIF-2α, VEGF	Pro-tumor—Resistance, angiogenesis	Standard: Bevacizumab Preclinical: Evofosfamide

## Data Availability

No new data were created or analyzed in this study.
